# Major depression and generalized anxiety disorder among HTLV-I/II infected former blood donors

**DOI:** 10.1186/1742-4690-8-S1-A70

**Published:** 2011-06-06

**Authors:** Anne M Guiltinan, Zhanna Kaidarova, Dee Behan, Cheryl Marosi, Sheila Hutching, Mandi Kaiser, Elane Moore, Deborah DeVita, Edward L Murphy

**Affiliations:** 1 Blood Systems Research Institute, San Francisco, CA 94118, USA; 2 American Red Cross Blood Services, Greater Chesapeake and Potomac Region, Baltimore, MD 21215, USA; 3 American Red Cross Blood Services, SE Michigan Region, Detroit, MI 48202, USA; 4 American Red Cross Blood Services, Southern California Region, Pomona, CA 91768, USA; 5 Oklahoma Blood Institute, Oklahoma City, OK 73104, USA; 6 University of California, San Francisco, CA 94118, USA

## Background

Other studies have reported high rates of depression and anxiety among human T-lymphotropic virus type 1 (HTLV-1) infected subjects, and have even suggested that HTLV-I causes psychiatric disease.

## Methods

We interviewed HTLV-I, HTLV-II and demographically similar HTLV seronegative blood donors with the Mini-International Neuropsychiatric Interview (MINI). Prevalences of major depression and generalized anxiety disorder in each group were calculated and compared to published U.S. population data. Adjusted odds ratios (aOR) and 95% confidence intervals (95% CI) controlling for educational achievement, alcohol intake and self-reported health status were calculated with multivariate logistic regression.

## Results

Major depression was diagnosed in 5 (5.4%) of 93 HTLV-I positive subjects (aOR = 2.19, 95% CI 0.63-7.55) and 17 (6.6%) of 256 HTLV-II positive subjects (aOR = 1.61, 95% CI 0.66-3.92), compared to 12 (2.1%) of 585 HTLV seronegative blood donors. The prevalence of major depression among infected subjects was comparable to the 6.7% prevalence in the U.S. general population. Generalized anxiety disorder was diagnosed in 5 (5.4%) HTLV-I positive subjects (OR= 2.32, 95% CI 0.74-7.26) and 12 (4.7%) HTLV-II positive subjects (OR = 1.65 95% CI 0.68-4.01), compared to 15 (2.6%) seronegatives and 3.1% in the U.S. general population.

## Conclusion

We observed slightly higher prevalence of major depression and generalized anxiety disorder among HTLV-I and HTLV-II subjects that was not significantly elevated after controlling for health status and other confounding variables. Comparison to U.S. population data suggested that these findings are in part explained by a “healthy blood donor effect” among our controls.

